# Clear cell sarcoma of the esophagus: A rare location

**DOI:** 10.1002/ccr3.2620

**Published:** 2019-12-22

**Authors:** Yosra Yahyaoui, Yosr Zenzri, Khalil Behi, Nadia Boujelbene, Amina Mokrani, Imen Abbas, Karima Mrad, Amel Mezlini

**Affiliations:** ^1^ Medical Oncology Department Faculty of Medicine of Tunis Salah Azaiez Institute El Manar University Tunis Tunisia; ^2^ Pathology Department Faculty of Medicine of Tunis Salah Azaiez Institute El Manar University Tunis Tunisia

**Keywords:** chemotherapy, clear cell sarcoma, esophagus, gastrointestinal

## Abstract

Clear cell sarcoma of the esophagus is very rare. The etiology of this neoplasm remains unknown. Confirmed diagnosis requires histopathology with immunochemistry and molecular study. CCS typically shows highly aggressive behavior with a high rate of local recurrence, metastases, and death from disease.

## Graphical abstract

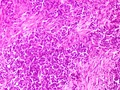

## 1 INTRODUCTION

Clear cell sarcoma (CCS) of the gastrointestinal tract is an aggressive neoplasm. The esophagus is an extremely rare location. We report the case of a 27‐year‐woman with CCS located in the esophagus. We analyze through this observation, the clinical, histological, and therapeutic characteristics of this entity.

Clear cell sarcoma is a rare soft tissue tumor derived from neural crest cells and melanocytes.^1^ It is an aggressive neoplasm that usually presents with metastatic disease and has a poor prognosis. Only a few cases of gastrointestinal CCS have been reported in the literature. We present here an extremely rare case of a CCS of the esophagus. To our knowledge, our case is the second case of CCS of the esophagus in the literature.

## 2 CASE REPORT

**Figure 1:**
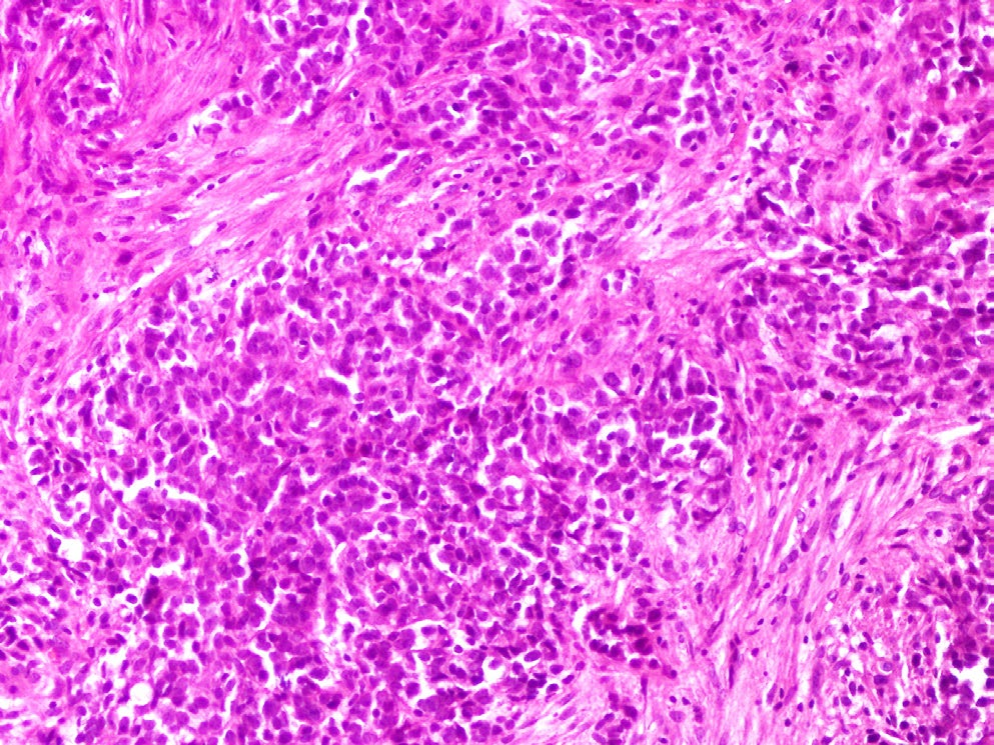
Compact nests and sheets separated by fibrous connective tissue (H&E, ×200)

**Figure 2:**
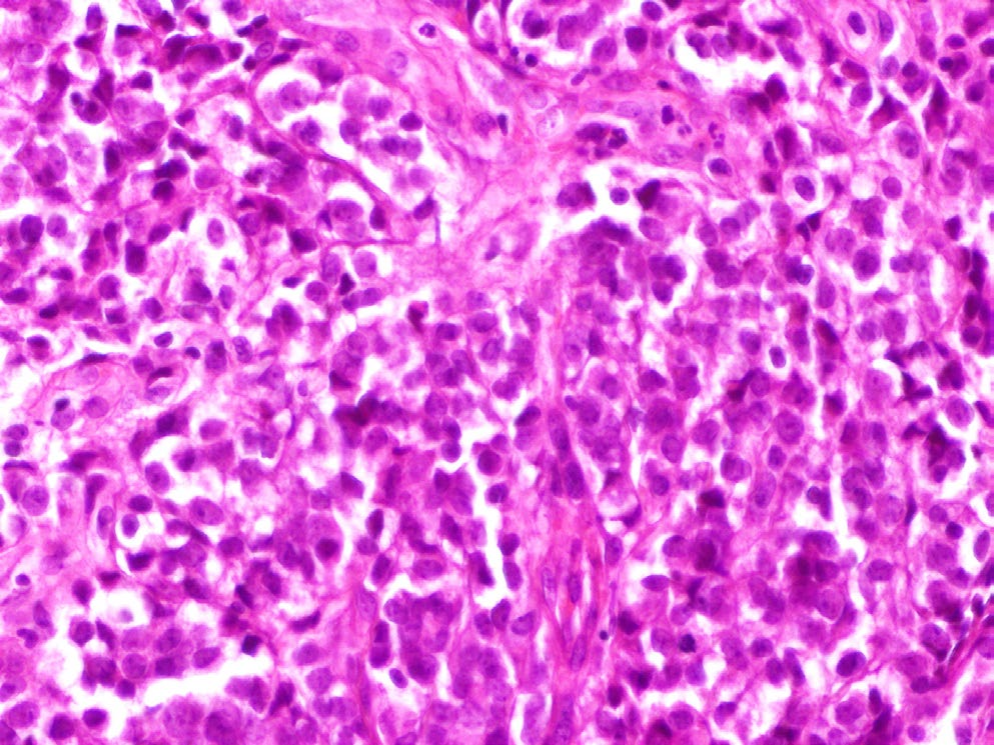
Oval to polygonal in shape cells with clear cytoplasm, vesicular nuclei with small nuceoli (H&E, ×400)

A 27‐year‐old woman with a history of retinoblastoma treated by enucleation of the left eye at the age of seven followed by a concurrent chemotherapy and radiation therapy was complaining of dysphagia to both solids and liquids and postprandial vomiting with a deterioration of the general status since three months. The physical examination was unremarkable. An upper gastrointestinal endoscopy was performed showing an ulcerogranulating lesion located at the middle esophagus spreading to 28 cm from 24 cm below the incisors. Endoscopic biopsies were performed. Histological examination showed small compact nests and sheets of neoplastic cells separated by fibrous connective tissue (Figure 1). Predominantly, the tumor was composed of oval to polygonal cells with clear cytoplasms and enlarged, irregular, hyperchromatic nuclei with nucleoli. Rare mitoses were observed (Figure 2).

**Figure 3:**
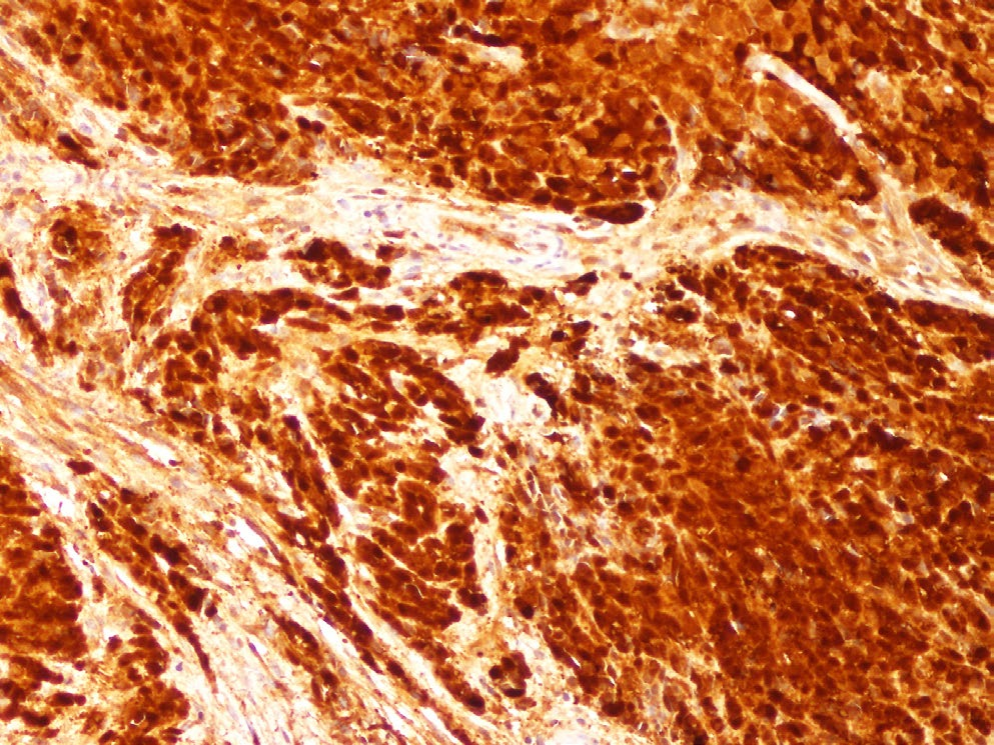
Diffuse positivity for S‐100 protein (×200)

Immunohistochemical staining showed that the tumor cells were positive for S‐100 protein (Figure 3), Sox10, RB1, CD99, and SMARCB1.

Stains for pan‐cytokeratin, HMB45, MelanA, Desmin, C‐kit, and DOG1 were negative.

Molecular study by RNA sequencing identified a EWSR1‐ATF1 fusion transcript. The histopathologic, cytogenetic, and immunohistochemical profile of this neoplasm was diagnostic of invasive CCS.

The abdominal, pelvic, and thoracic computed tomography showed a locally advanced tumor of the esophagus invading the carina, trachea, and the aorta. Lung metastases were observed. The patient underwent seven cycles of doxorubicin. CT scan showed stable disease. She had a stable disease for a total duration of seven cycles of her treatment. She developed chemotherapy‐related cardiotoxicity after doxorubicin worsening her left ventricular function. The patient died before starting ifosfamide regimen.

## 3 DISCUSSION

Gastrointestinal CCS is a very rare sarcoma subtype with few cases described in the literature. It occurs most often in tendons and aponeuroses affecting adolescents and young adults.^2^ It is characterized by highly aggressive clinical behavior with a high risk of recurrence and metastatic disease. The clinical presentation is often associated with intestinal obstruction, abdominal mass on imaging, abdominal pain, or nonspecific symptoms such as anemia, vomiting, anorexia, weight loss, hematemesis, or rectal bleeding.^3^ It should be noted that our patient had a medical history of unilateral retinoblastoma treated at the age of seven. Long‐term survivors of hereditary retinoblastoma face an increased risk of developing bone and soft tissue sarcomas due to past radiation treatment and genetic susceptibility. Soft tissue sarcomas are frequently observed in hereditary retinoblastoma accounting for 12% up to 32% of all second cancers.^4^ In our case, the retinoblastoma gene was expressed in tumor cells, which would not be in favor of a hereditary retinoblastoma. Furthermore, radiation therapy can increase the risk of clear cell sarcoma.^5^ Our patient was treated by concurrent chemotherapy and radiation therapy for the retinoblastoma at the age of seven.

The diagnosis of CCS is difficult as it requires a combination of morphological, immunohistochemical, and molecular studies. Histological examination usually shows a tumor formed by oval or spindle‐shaped cells organized in alveolar solid or flux‐like reticulum.^6^ Immunohistochemistry is mandatory for the diagnosis. Immunohistochemical staining shows that the tumor cells are positive for S‐100 protein in all cases. Melanoma‐related markers can be positive.^7^ The absence of melanocytic differentiation, the S‐100 protein and SOX10 protein positivity suggest that CCS could originate from primitive neural crest cells.^8^ In most cases of CCS, this neoplasm is associated with the reciprocal translocation t (12;22)(q13;q12) resulting in fusion of the EWSR1 gene and the ATF1 gene.^9^ These translocations make the difference between CCS and malignant melanoma which has similar histological appearance to clear cell sarcoma.^10^

Furthermore, gastrointestinal (GI) CCS shares common characteristics with the gastrointestinal neuroectodermal tumor (GNET). Both GI CCS and GNET are rare. Morphological features make the difference between GI CCS and GNET. GI CCS is characterized by nests and fascicles of relatively uniform polygonal, epithelioid, and spindle cells, separated by fibrous septa. Rounded vesicular nuclei with prominent nucleoli are observed in tumor cells. Solid, nested, and fascicular architecture are observed in GNET. Pseudoalveolar and rosette‐like arrangements are also noted in GNET. Melanosomes and premelanosomes are often observed in GI CCS. They are absent in GNET.^11^

The presence of osteoclast‐like multinucleated giant cells is the most helpful distinguishing feature of GNET.^12^

In GI CCS, EWSR1 gene alteration is usually fused with the ATF1 gene (61.5%). In GNET, EWSR1 gene alteration is fused with the ATF1 gene (32.8%) and with the CREB1 (17,2%).^11^ The relationship between CCS‐GIT and GNET continues to be debated.

In CCS, metastases in the lymph nodes and lungs are frequent, as well as local recurrence. This tumor is known to be resistant to chemotherapy and radiation therapy. Consequently and regarding to the scarcity of the disease, there is no established regimen for clear cell sarcoma. The cornerstone of multidisciplinary treatment is surgery. In fact, in another reported case of a clear cell sarcoma of the esophagus, the patient has been treated by surgery alone and is still alive 14 months after undergoing resection with no evident signs of recurrence.^13^ In our case, the tumor was unresectable because of vascular, tracheal invasions, and lung metastases. The 5‐year survival rate of CCS was reported to be 52%.^14^

Due to the resistance to classical chemotherapy, trials of molecular targeted therapies are ongoing. The CREATE trial tested the efficacy and safety of the tyrosine kinase inhibitor crizotinib in patients with advanced or metastatic CCS. Twenty‐six out of the 28 patients had MET (+) disease. The PFS with crizotinib in MET+ CCS was similar to results achieved with doxorubicin.^15^

The NCT00557609, a phase 2 trial, has shown that disease control was obtained in 36% patients with tivantinib, a MET inhibitor.^16^ Mir et al reported a case of metastatic CCS with myocardium metastasis. He received sorafenib with a clinical benefit and reduction in the lesion size. The progression‐free survival was 8.2 months.^17^

Few reports on the efficacy of immunotherapy have been reported. The NCT01445379, a phase 1 clinical trial, studied CTLA‐4 antibody and immune checkpoint modulator ipilimumab in pediatric patients with advanced cancers included two children with metastatic CCS. One of them experienced stable disease for a total duration of six cycles of this treatment.^18^ The rarity of CCS makes modulation of targeted therapies hard to run.

## 4 CONCLUSION

Gastrointestinal CCS is an aggressive soft tissue sarcoma. The esophagus is an extremely rare location. Pathological examination and immunohistochemistry are necessary for a conclusive diagnosis. Multidisciplinary approach is mandatory. CCS frequently relapses and has distant spread. The prognosis remains poor.

## CONFLICT OF INTEREST

None declared.

## AUTHOR CONTRIBUTIONS

YY: devised the concept. YY, ZY, and BK: participated in design and definition of intellectual content. ZY and BK: participated in literature search. YY, ZY, BN, BK, MA, AI, MK, and MA: participated in manuscript preparation. YY, ZY, and BN: edited the manuscript. YY: served as the guarantor.
